# FPGA Based Adaptive Rate and Manifold Pattern Projection for Structured Light 3D Camera System †

**DOI:** 10.3390/s18041139

**Published:** 2018-04-08

**Authors:** Muhammad Atif, Sukhan Lee

**Affiliations:** Intelligent Systems Research Institute (ISRI), College of Information and Communication Engineering, Sungkyunkwan University, Suwon, Gyeonggi-do 440-746, Korea; m.atif@skku.edu

**Keywords:** structured light 3D camera system, hardware synchronization, adaptive frame rate pattern projection

## Abstract

The quality of the captured point cloud and the scanning speed of a structured light 3D camera system depend upon their capability of handling the object surface of a large reflectance variation in the trade-off of the required number of patterns to be projected. In this paper, we propose and implement a flexible embedded framework that is capable of triggering the camera single or multiple times for capturing single or multiple projections within a single camera exposure setting. This allows the 3D camera system to synchronize the camera and projector even for miss-matched frame rates such that the system is capable of projecting different types of patterns for different scan speed applications. This makes the system capturing a high quality of 3D point cloud even for the surface of a large reflectance variation while achieving a high scan speed. The proposed framework is implemented on the Field Programmable Gate Array (FPGA), where the camera trigger is adaptively generated in such a way that the position and the number of triggers are automatically determined according to camera exposure settings. In other words, the projection frequency is adaptive to different scanning applications without altering the architecture. In addition, the proposed framework is unique as it does not require any external memory for storage because pattern pixels are generated in real-time, which minimizes the complexity and size of the application-specific integrated circuit (ASIC) design and implementation.

## 1. Introduction

Non-contact optical 3D scanning has rapidly evolved due to the availability of extreme computation power, precise measurement, high scan speeds, and advancements in optics [1]. Several approaches have been presented in the literature for obtaining a depth map, including stereo vision [2]; time-of-flight (ToF) [3]; depth from focus/defocus [4]; and structured light [5,6,7,8,9]. In stereo vision, two imaging sensors are used to capture images with a known baseline length and orientation. Common features are extracted from both images using techniques such as Scale Invariant Feature Transform (SIFT) [10] or Speeded Up Robust Features (SURF) [11], which are used to make pixel correspondence, and depth is computed by triangulation.

One of the major limitations of stereoscopy is that this approach fails to obtain depth information of the texture-less surface despite being by far the most frequently used technique. In time-of-flight cameras, a light signal is sent from the transmitter and a receiver is used to detect the reflected signal. The depth map is computed from the time taken by the signal to reflect back from the object surface [12]. One of the renowned time-of-flight based 3D imaging sensors is Microsoft Kinect-v.2 [13], which provides a depth map of a scene and helps in many fields where accuracy requirement is modest. This approach is rather limited in applications as it gives a sparse depth map and also cannot differentiate between inter-reflected and original signals. In the depth from focus/defocus approach, several images are captured at different focal lengths. The amount of blur or blur size is computed from each image and the camera model is used to compute a depth map. This method is prone to shadow as it induces artificial blurring. Structured light 3D camera systems are composed of cameras and projectors, where a sequence of temporally or spatially multiplexed pattern signals is projected on a scene by a projector and its image is captured by a camera. A depth map is obtained by decoding pattern signals from the captured image for triangulation. Structured light 3D camera systems are widely used in the field of robotics [14,15], inspection of printed circuit board (PCB) [16], industrial automation [16,17,18], saving cultural heritage [19], examination of arc welding pools [20], object recognition [21], and dental surgery [5].

Quality of point cloud and scan speed of the structured light 3D camera systems depend on obtaining a good quality point cloud in the presence of surfaces with large reflectance variation, projector–camera synchronization, and changing the scan speed adaptively in accordance with the scan requirements. 

A real-time IR based structured light 3D camera was suggested by Lee et al. [22] and Field Programmable Gate Array FPGA is used to generate the patterns, and the patterns are projected with a specific frame rate which means that the projection frame rate must be the same as that of the camera frame rate. Wang et al. [23] suggested an architecture where a depth frame rate of 120 fps was achieved with an external trigger generated from the projector. A Digital Light Processing (DLP) projector kit was used to synchronize the projected frames to the captured frames [24] to reconstruct depth information accurately [25]. Zhang et al. [26] suggested a 30 fps depth frame rate by the synchronized capturing of three phase shifted fringe patterns projected with a DLP projector. These systems work perfectly for a single camera exposure setting and capture the same scene with two camera exposure settings, with the patterns needing to be reprojected.

Exposure settings of the structured light 3D camera systems are important [27] in terms of single or multiple capture and camera exposure time, multiple exposures are required to obtain the depth information if the scene contains surfaces with large reflective variation. In conventional projector camera synchronization systems, the trigger for the camera is generated once for each pattern [22] to capture the frame with a single exposure time, and multiple projections are required to capture the scene with multiple camera exposures [28]. Multi-exposure, phase shifting, and the High Dynamic Range (HDR) techniques are used to measure depth information of shiny and dark surfaces [29,30]. In this paper, we propose a method where the frames can be captured multiple times within the projector exposure time, and the position and number of camera triggers are adaptive and flexible in terms of position.

Synchronization of the projected patterns to the captured frames is necessary to achieve an accurate point cloud, and synchronization can be made through the software or hardware [31]. Hardware synchronization requires additional hardware [25] or the vertical synchronization signal of the Video Graphic Array (VGA) frame can be used to capture the frame synchronously [17]. The quality of the point cloud obtained through the hardware synchronization is better when compared to the software synchronization due to the asymmetric exposure time of the camera for each pattern. FPGA has been used as the external hardware to generate the patterns and trigger the camera for structured light systems [24,32,33,34], but these projector–camera systems are a perfect example of a fixed rate projection system where the camera frame rate and projector screen refresh rate must be the same as the system cannot synchronize if the projector and camera have different screen refresh and frame rate, respectively. A new hardware approach to synchronize the projector–camera system is the use of DLP projector kits, which projects the patterns with high speed and generates the trigger for the camera [12,17,25,28,35]. These systems fail to generate the trigger multiple times to capture scenes with different exposure settings which are necessary in situations where multiple exposures are required to capture scenes with large reflective variations. In this paper, we implement a framework on FPGA which synchronizes the camera and projector system for a matched and/or mismatched camera frame rate and projector refresh rate. Three synchronization modes are implemented by which a machine vision camera with any frame rate and commercial projector can be synchronized. The proposed implementation projects the patterns adaptively as well as generates the camera trigger flexibly. 

The scan speed of a structured light 3D camera system, the precision, and density of the point cloud are interlinked; for high scan speed applications, the quality of the point cloud is compromised and vice versa. High scan speed is required in industrial automation where objects are moving [36,37,38] and large vibrations, accuracy, and precision are required in critical applications such as dental surgery and PCB inspection [39]. High scan speed can be achieved by reducing the number of patterns such as hybrid [38], one shot [40], or color-coded [41] patterns used to capture moving objects [36], resulting in the accuracy of the output being compromised [42]. The framework implemented in FPGA is flexible in terms of pattern projection. Patterns can be selected based on scan application, e.g., for high scan speed applications [43], hybrid patterns can be used and, for high precision applications, Hierarchal Orthogonal Coded (HOC) patterns [44] or Gray Code Inverse (GCI) [45] patterns can be used. The change in the type of the pattern is completely flexible, which does not require any changes to the architecture of the camera-projector system. This makes the system more flexible for use in different kinds of applications to fulfill different scan speed requirements. FPGA is selected as an external hardware module to project the different structured light patterns flexibly and to synchronize the camera at any frame rate. The proposed method is unique in two aspects: pattern pixels are generated in real-time without using any external memory, and the frame adaptation feature can be used for any kind of pattern.

The contributions of this paper are as follows:

- Real-time pattern pixel generation without using additional external memory to the FPGA;
- A single platform for multiple types of structured light patterns projection;
- Improvement in the quality of the point cloud for surfaces with large reflectance variations; and
- Hardware-based synchronization framework for a structured light 3D camera system for mismatched projector screen refresh and camera frame rates.

The rest of the paper is organized as follows: the structured light 3D camera is discussed in Section 2; the framework is proposed in Section 3; the experimental results are presented in Section 4; and the paper concludes in Section 5. 

## 2. Structured Light 3D Camera

In the structured light system, stripe patterns are projected from a projector and an imaging sensor is used to capture them. Stripe patterns are deformed due to the object shape, which can be used to compute the 3D geometry. Structured light techniques have been in the limelight generally due to recent advancements in digital technologies and the availability of high-speed projection and capturing capability [46]. Structured light 3D camera systems are composed of the illumination source and a sensor to receive the light reflected from the surface, which is why structured light 3D camera systems are known as active systems. Applications of these structured light systems are very wide in the fields of health [5], industry [17], agriculture [47], reverse engineering, entertainment, robotic vision [15], and geology. High-speed 3D map acquisition, its noncontact [42] nature, and high precision make structured light techniques suitable and highly adaptable in the fields of medicine, reverse engineering, processing and manufacturing, remote monitoring, profiling, etc. 

In [48], a robotic manipulator equipped with a structured light 3D vision sensor was developed for quality control in the manufacturing industry. In [47,49], a structured light system additionally equipped with multiple cameras was presented for use in the inspection and clearance of railway tunnels and 3D reconstruction of plants, respectively. In [50], sinusoidal fringe patterns in the infrared range were used to formulate a 3D model of the face and body. In [51], a structured light vision system was developed for automated welding in the shipbuilding industry. Several other applications including dental scanning [52], underwater surface profiling [53], and eye on hand for modular manipulator [54,55,56] have also been reported in the literature. An IR based adaptive baseline based camera was designed for a robotic arm to capture objects at different distances [57]. One of the major advantages of the structured light 3D camera systems is high-speed precise measurement in the industrial environment where the stereovision system cannot perform well due to poor illumination conditions.

High scan speed structured light 3D cameras are required for the inspection of moving objects [58], e.g., to inspect car parts on the assembly line [57], which becomes difficult due to the movement of the assembly line and the vibration in the hanging part. The high scan speed of the structured light 3D camera system can achieve this through projecting patterns with high projection speed [12], reducing the number of patterns [40], using color-coded patterns [41], and performing the decoding process on the Graphic Processing Unit (GPU) [58].

### 2.1. Structured Light Patterns

Structured light patterns are the sequence of the stripes that are projected on the scene and are captured by the camera to reconstruct 3D information. Pattern encoding can be classified in two ways: spatial and temporal coding, spatial coding generates a spatially distributed pattern to represent individual locations with unique codes, while temporal coding generates a successive projection of multiple patterns in time for the same purpose. In addition, hybrid coding combines spatial and temporal coding to take advantage of both. For high scan speed applications, spatial encoding patterns are used to capture the moving objects, and for precise measurement, temporal patterns are used [59]. In this paper, we implemented temporal and hybrid coding with binary patterns to obtain the depth information.

**Figure 1:**
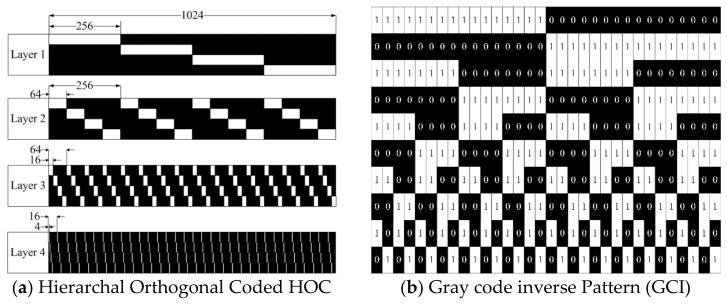

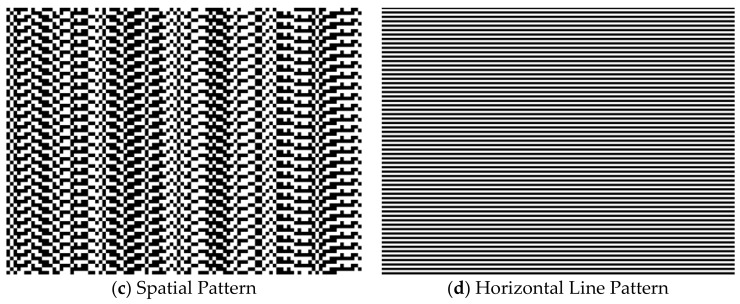
Structured light patterns projected through Field Programmable Gate Array (FPGA): (**a**) Hierarchal Orthogonal Coded patterns (HOC); (**b**) Gray Code Inverse (GCI) patterns; (**c**) Spatial patterns; and (**d**) Horizontal Line patterns, (**c**,**d**) combined are hybrid patterns that are used to reconstruct depth information.

HOC patterns are robust structured light patterns, which are discovered due to the degradation of precision and robustness in conventional structured light patterns that appear due to overlapping multiple stripe code. Stripe patterns are separated by making the patterns orthogonal. The length of the code is reduced by arranging the orthogonal hierarchically. In the encoding process, the “f” length of the code signals are divided into a few layers “L”, and each layer includes “H” orthogonal codes recursively, as shown in Figure 1a [44]. Gray code inverse patterns are temporal binary patterns that are commonly used to compute depth. Here, the 5-bit gray code is shown in Figure 1b. Hybrid patterns are used for the high scan speed applications. Hybrid patterns are a combination of temporal and spatial patterns. In hybrid patterns, two temporal patterns and spatial patterns are used to decode the region shown in Figure 1c,d.

### 2.2. Patterns Decoding

**Figure 2:**
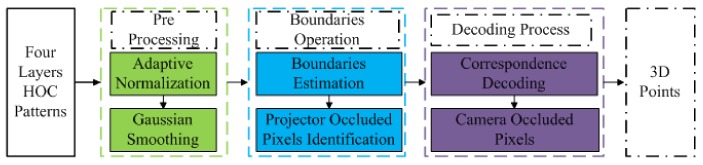
Flow diagram of Boundary Inheritance Codec decoding process.

Boundary Inheritance Codec (BIC) [60] is a process of decoding the HOC patterns accurately to obtain the depth information. The BIC decoding process is shown in Figure 2. The decoding process is divided into three main sections: pre-processing, boundary operation, and decoding mechanism. In the first step, the captured signal is converted into the canonical form where normalization [61] and Gaussian smoothing is carried out; in the second step, stripe boundaries are estimated along with projector occluded pixels [62]; and, in the third step, correspondence is made between the detected boundary to the projected stripe boundary. After obtaining the stripe boundaries, the stripe boundaries interpolate and outliers are removed [63]. Gray Code Inverse (GCI) patterns are more robust to noise to obtain dense point clouds [45]. Hybrid patterns are decoded by using the boundary based region based searching method presented in [64].

## 3. Proposed Framework

The synchronization and adaptive framework implemented in the FPGA are discussed in this section. Initially, the implementation block diagram is presented, and further implementation of the framework and the adaptive mechanism are presented afterwards.

### 3.1. Block Diagram

**Figure 3:**
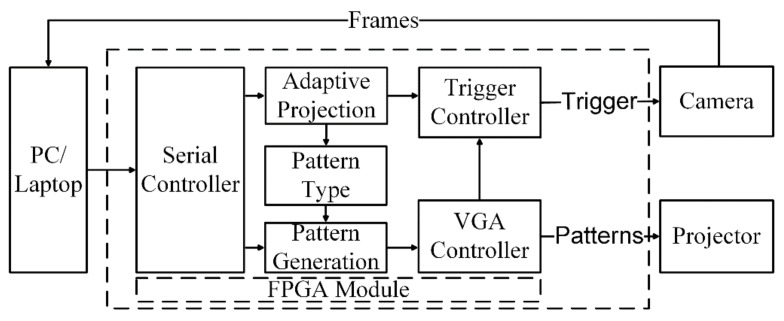
Block level representation of the complete architecture implemented on FPGA which contains the synchronization, adaptive rate pattern generation and camera trigger controller module.

The complete implementation of the system consists of six sub-modules, which are shown in Figure 3. The serial controller module establishes a communication link between the computer and the FPGA controller module. The projection command is sent to the controller which obtains four important pieces of information: (1) type of the pattern; (2) frequency of the projection; (3) positions of the trigger for the camera; and (4) the number of camera triggers for single or multiple camera exposures. The frequency of the pattern projection and the trigger settings for the camera are determined through the exposure settings of the scene. The frequency of the pattern projection is defined as the number of times to project the same pattern. For a higher projector refresh rate, the same pattern is projected and, for a lower projector refresh rate, the same patterns are captured multiple times, or the camera is triggered multiple times.

As the projection command is received by the serial controller, the pattern generation module switches from idle to the pattern projection state. Initially, a few frames are not projected on the scene as those are required to determine the resolution and refresh rate, so it is desirable to keep the projector alive while patterns are not being projected. This is represented as an idle state, where the idle state is defined as a stream of pixels with zero intensity being fed to the projector. The pattern type module generates the pixels of the pattern to make the VGA frame. The VGA controller module receives the pixels generated by the pattern generation module and generates the vertical and horizontal synchronization signal of the VGA frame. The complete frame is sent to the projector after receiving pixels from the pattern generation module encapsulated by the VGA controller along with the synchronization signals. The camera trigger module requires three signals to generate the trigger for the camera to capture the frame synchronously.

### 3.2. Serial Controller Module

A universal asynchronous receiver and transmitter (UART) is implemented in the FPGA module to communicate with the computer through the RS232 protocol. A universal serial bus (USB) to serial converter is used to connect a laptop/embedded platform/computer to the FPGA. This module provides a communication bridge between the machine and the FPGA. This module translates the commands from the machine to the pattern generation module.

### 3.3. Pattern Type Selection Module

The pattern generation module is one of the core modules which generates pixels of the structured light patterns. All kinds of patterns can be generated from the FPGA to project on the scene to compute depth; in this paper, we present three patterns that can be projected: HOC, GCI, and hybrid. Implementation of the pattern generation module is novel in terms of generating the pattern pixels. The uniqueness of the proposed pixel generation method is that pixels are generated in real-time without using any external memory to save the patterns. In conventional pattern generation mechanisms, the patterns are stored in the memory and then pixels are generated by reading the saved images, which requires additional memory. 

**Figure 4:**
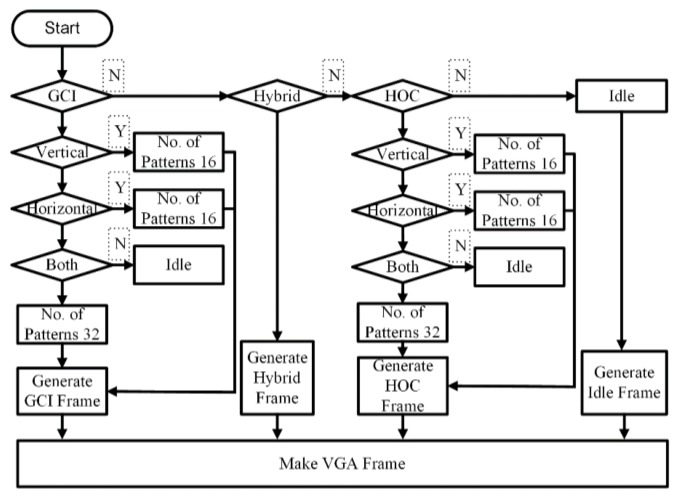
Internal flow diagram of the pattern selection module and selection of a procedure for patterns projection.

In the proposed method, the pattern pixels are generated in real-time, which minimizes the utilization of the logic blocks of the FPGA and reduces the complexity and size of the application-specific integrated circuit (ASIC) design and implementation. Details of each pattern generation are discussed in the latter part of this paper. A complete flow diagram of the pattern generation is presented in Figure 4. For HOC and GCI, patterns can be projected in a vertical and/or horizontal direction depending upon the application and configuration of the camera and projector. For high precision measurement, vertical and horizontal patterns can be used similarly for the lateral configuration of the camera, and projector vertical patterns are used and vice versa for the other.

### 3.4. Adaptive Rate Pattern Projection Module

The adaptive rate pattern generation module is one of the intelligent modules of the complete framework. This module synchronizes the camera and projector by using the camera exposure settings and projector screen refresh rate. The screen refresh rate of the commercial video projectors is fixed, and the fps of the machine vision embedded cameras is flexible. Lowering the exposure time will result in a higher camera fps and vice versa for a higher exposure time. The adaptive frame rate pattern generation is crucial, where the projector refresh rate does not match the camera frame rate. A camera with a higher frames per second (fps) can be used with a fixed rate projector by changing the exposure time of the camera. Three conditions can appear for the projector refresh rate and camera fps: (1) the projector and camera refresh rate are identical; (2) the projector refresh rate is higher than the maximum camera fps; and (3) the camera fps is larger than the projector screen refresh rate. All possible cases are implemented to obtain a good quality point cloud. During the discussion of the paper, we used a 60 Hz projector screen refresh rate, which means that the exposure time for one frame was 16.67 ms.

#### 3.4.1. Synchronization for Identical Projection and Camera Frame Rate

**Figure 5:**
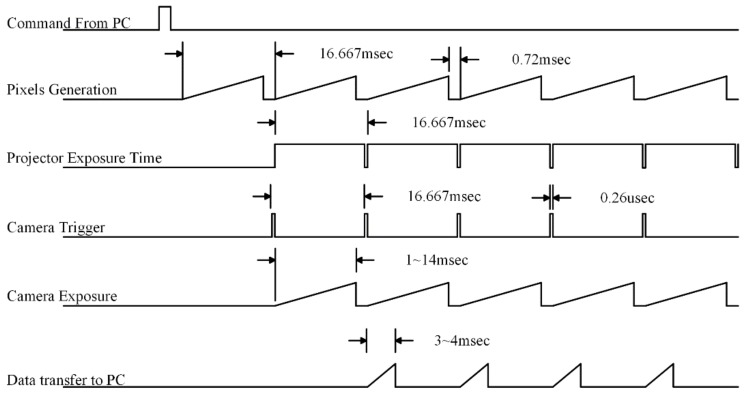
Timing diagram for projector screen refresh rate matched with camera frame rate.

A commonly presented case of hardware synchronization is where the camera frame rate and projection refresh rate are the same [58]. The timing diagram of this particular case is presented in Figure 5. The projector exposure time was 16.67 ms. As the projector starts the exposure, the FPGA will trigger the camera to capture the frame, so the exposure time of the camera depends upon the scene conditions, but the maximum allowable exposure time will be 16.67 ms. The camera opens its shutter and acquires the light until the camera exposure time is reached. After the integration process, the frame is sent to the computer. This procedure keeps going until the last pattern [24]. In this case, no additional frames are projected and no additional frames are captured.

#### 3.4.2. Adaptive Synchronization for Projection Rate Larger than Camera Frame Rate

**Figure 6:**
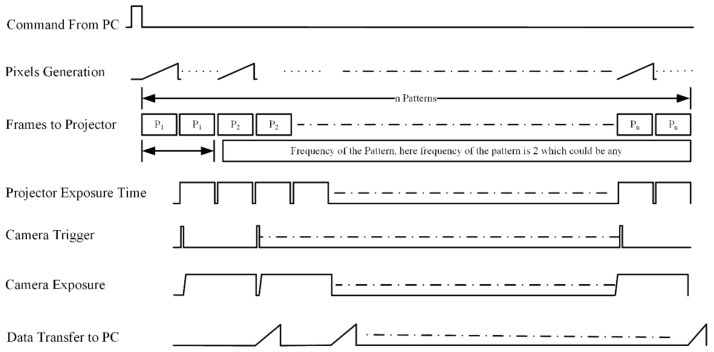
A timing diagram for adaptive frame rate pattern projection for projector camera synchronization when projector screen refresh rate is higher than the camera frame rate.

Synchronization between the camera and projector becomes difficult when the projector screen refresh rate is higher than the camera frame rate. This appears due to a lower camera frame rate, which usually appears in high-resolution cameras such as a Grasshopper industrial vision camera [65]. Synchronization of such systems can be done through software [31], but that does not guarantee the quality and precision of the point cloud due to asymmetric camera exposure time. The timing diagram of the implementation of the system is shown in Figure 6, which shows that the frequency of projection of the same patterns is determined through the camera exposure time. The larger the camera exposure time, the higher the frequency of the projection frame will be. The same frame will be projected until the exposure time of the camera, and one of the limitations of the system is that the projector exposure time will be in a multiple of 16.67 ms as the projector frame rate is fixed to 60 Hz. This can be seen clearly when the same pattern is projected twice, which can be projected n times, as that is how long the camera will be exposed to that frame.

In this example, the maximum fps is half of the projector screen refresh rate, e.g., 60 Hz projector screen refresh rate and 30 fps camera frame rate, and the maximum allowable adaptive framework can synchronize up to 0.3 fps of the camera.

**Figure 7:**
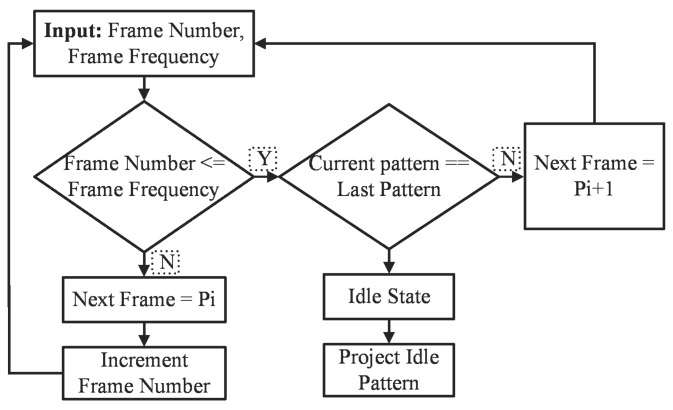
Block diagram of adaptive frame rate projection controller module in FPGA.

The implementation of the adaptive frame rate mechanism is presented in Figure 7. The adaptive frame rate controller module keeps generating the same frame until the frequency of the pattern is achieved. Here, Pi shows the particular frame pattern; if the frame number matches the frequency of the pattern, the pattern generation module keeps generating the same pattern, and this mechanism keeps going until the last pattern.

#### 3.4.3. Adaptive Synchronization and Multi-Frame Capturing for Lower Projection Rate than Camera Frame Rate

This is one of the state of the art methods to obtain an accurate and dense point cloud of the surface with large reflectance variation by projecting the patterns once on the scene. The projection frame rate of the commercial video projectors is fixed, and they have a particular pixel clock speed to accept the frames to be displayed. In this paper, we used a pattern resolution of 1024 × 768 with a refresh rate of 60 Hz, which requires a pixel clock of 65 MHz. Similarly, the camera used in this experiment was a Chameleon-3 from point grey [66], which offers a frame rate of 149 fps. In this paper, we proposed a method to utilize the high frame rate of the camera to obtain the dense point cloud by utilizing the larger exposure range of the camera. The density, precision, and quality of the 3D point cloud captured by a structured light 3D camera system depends upon the optimal exposure settings. In [67], two exposure times were suggested for the 3D reconstruction of a scene with different albedos and criteria were established to check the pixel conditions for the reconstruction of the 3D information, so it was necessary to project the same patterns twice and capture the frames for 3D reconstruction.

**Figure 8:**
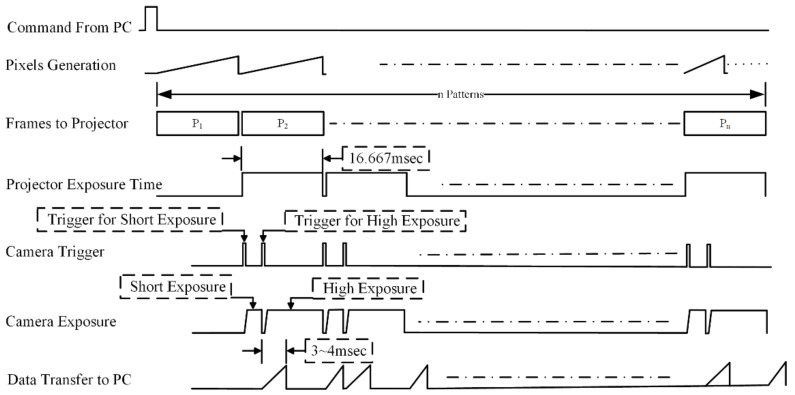
Timing diagram of multi-camera exposure in single projector exposure. Refer to the camera trigger signal for multiple capturing of low and high exposures within a single camera exposure setting.

Multiple exposures are required to obtain the point cloud from surfaces with different reflectance characteristics [17]. In this paper, we proposed a state of the art hardware approach to capture the same frames with different camera exposure settings without re-projecting the patterns. The embedded projector [12] triggers the camera once at the start of each frame, which indicates that patterns must be reprojected to capture different camera exposure times. The adaptive frame pattern projection mechanism can be performed to capture the same frame as many times as required without reprojecting the patterns, which reduces the projection and capture time to half by triggering the camera multiple times. To obtain the points from the highly reflective surface, the camera exposure time should be short and a larger exposure time is required to capture the depth information from the low reflective surfaces that absorb the light and where the reflection of light is very low. A timing diagram for the mismatched camera and projector frame rate is shown in Figure 8.

The camera is triggered twice: once to capture the frame for a lower exposure time, and once for the higher exposure time. The projector exposure time is fixed to 16.67 ms, so both the frames are captured within this exposure time. As the frames are captured multiple times, the point cloud obtained from two different exposure settings needs to be merged, as shown in Figure 8. A state of the art method was proposed to merge the point cloud, which minimized the outliers so a clean point cloud could be obtained.

**Figure 9:**
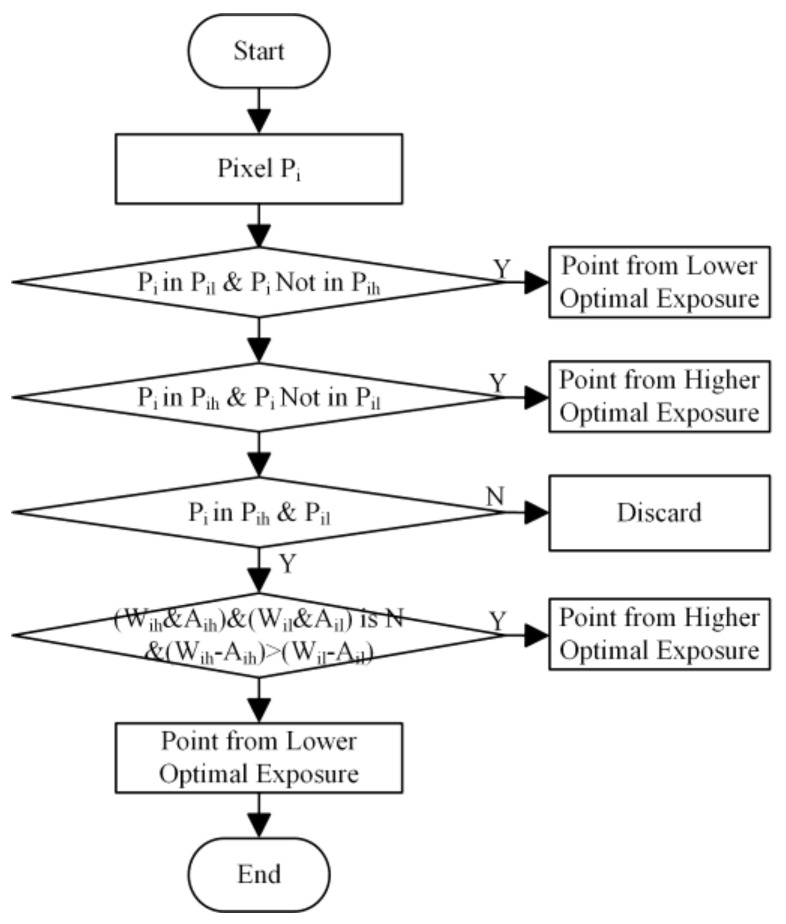
Flow diagram of the merging of two point clouds obtained from the two different exposure settings of the structured light 3D camera.

Point clouds obtained with a lower optimal exposure time and higher optimal exposure time need to be merged into a single point cloud. The process of merging the two point clouds is shown in Figure 9. Here, Pi, Pil, and Pih represent the particular pixel, and a point obtained by a lower and higher exposure time, respectively. A 3D point recovered from only one of the exposure times is unconditionally selected. If a point is recovered from more than one exposure time, pixel with higher intensity difference of white and ambient will be selected, e.g., if the intensity difference of pixel from larger exposure (Wih–Aih) is higher than the intensity difference from shorter exposure (Wil–Ail), then the point obtained through larger exposure will be selected and vice versa. Here, Wih, Aih, Wil, and Ail represent the intensity of the pixel from white frame obtained with a high exposure time, an ambient frame with a high exposure time, a white frame with a low exposure time, and an ambient frame with a low exposure time, respectively. White and ambient frames are those obtained by projecting all one and all zero pixels, respectively.

### 3.5. Pattern Generation Module

The pattern generation module is controlled by the adaptive frame rate module and the pattern type module. One row of each pattern is stored in the register, which is initialized as the board is powered up and starts generating the pattern pixels. Three binary pattern generation modules were implemented to generate the pixels for the projection. Pattern generation module implementation is unique in terms of resource optimization as no external memory is used to store the patterns and pattern pixels are generated by making the logical representation of the patterns. A more detailed implementation is discussed below.

#### 3.5.1. Hierarchal Orthogonal Coded (HOC) Pattern Generation

**Figure 10:**
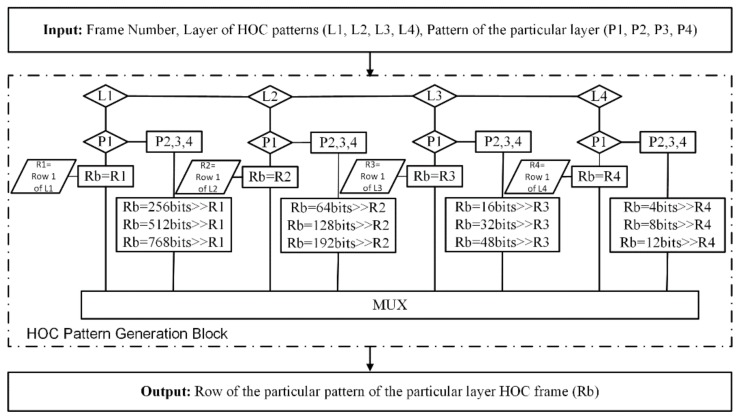
HOC patterns generation module generates patterns from the first row of each layer of HOC patterns by using the register shift logic.

In this implementation, four layer HOC patterns were selected, which means that each layer will have four patterns and each layer has 4^L codes and these codes are orthogonal to each other. The pattern resolutions were 1024 × 768, which means that 256 unique codes will be projected on the scene to compute depth. Here, “L” shows the layer of HOC patterns. The binary intensity value of the first row each pattern stores in the four registers. Later, these registers will be used to make the 16 HOC patterns. To generate the pattern of layer “L”, the binary stored values are shifted with the same number as that of the width of the stripe of that layer, e.g., to generate a second pattern of the first layer, the pixels of the first layer register is shifted with 256 pixels. The implementation of this system is shown in Figure 10. In Figure 10, “L” represents the layer, “P” represents the patterns, and R1, R2, R3, and R4 represent the four registers of the HOC patterns. “Rb” is the buffer register which stores the binary values of the current pattern being projected. This is the unique way by which the patterns are projected without having to be saved in the external memory. Instead of storing the patterns, the patterns are generated in real time without any delay.

#### 3.5.2. Gray Code Inverse (GCI) Pattern Generation

**Figure 11:**
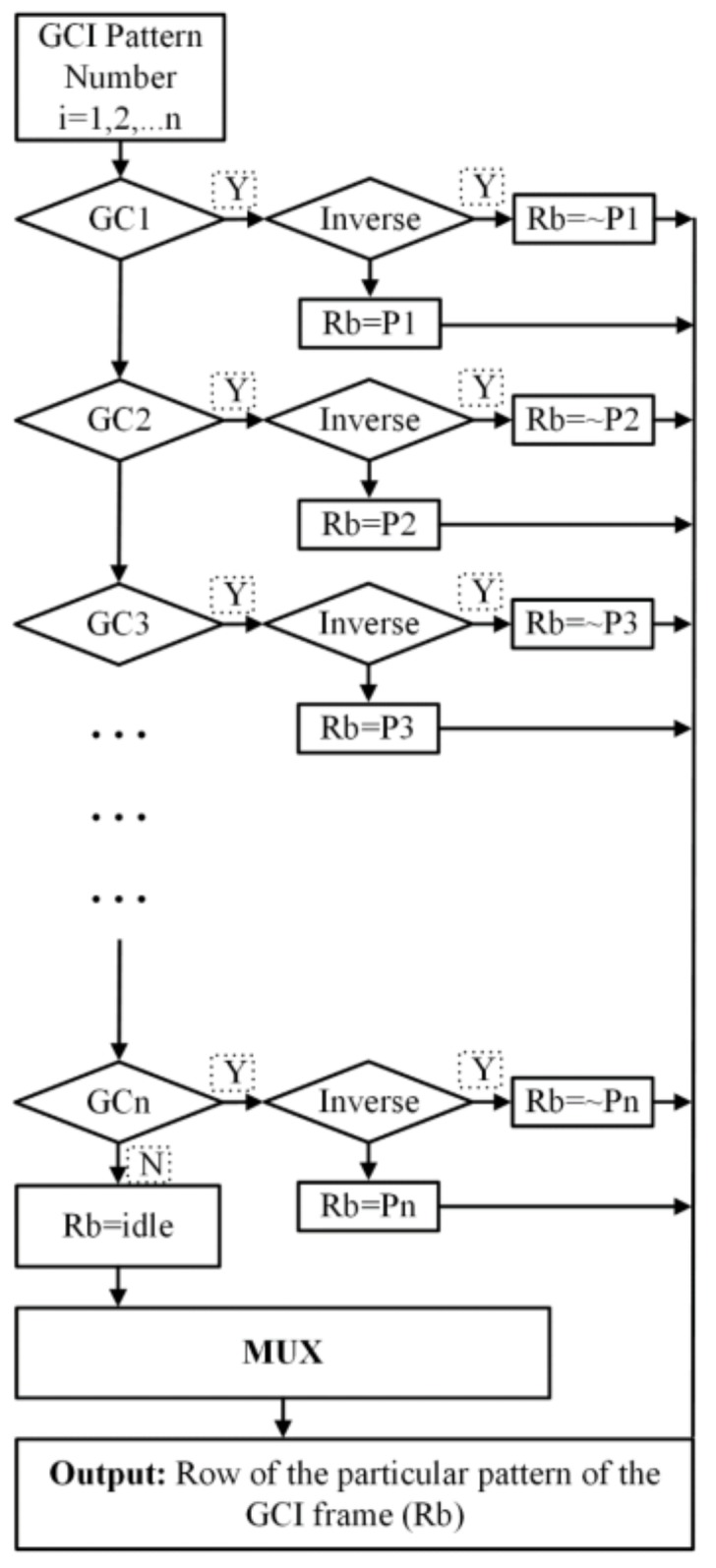
GCI patterns generation module.

GCI has commonly used patterns for a structured light camera-projector system due to the robustness, and it has also been implemented to project patterns in a vertical, horizontal, or both directions simultaneously. Here, we used 8-bit GCI patterns, where, for each pattern direction, there will be 16 patterns to be projected and 32 patterns for both. The pattern resolution is the same as that of the HOC patterns (1024 × 768), so there will also be 256 unique codes projected on the scene. Eight registers were initialized with the binary intensity of the first row of each of frame. Here, P1, P2, …, Pn are the eight (*n* = 8) registers which are initialized, and “Rb” represents the temporary buffer used to make the projection patterns. This module runs on pixel clock, on each clock cycle, one value is picked and transferred to the multiplexer. To project a pattern in sequence for each frame, the particular row is assigned to the buffer “Rb”. The inverse of GC is projected by inverting the stored registers as shown in Figure 11. This is the mechanism by which the pixels are generated in real-time for a complete frame.

#### 3.5.3. Hybrid Patterns Generation

**Figure 12:**
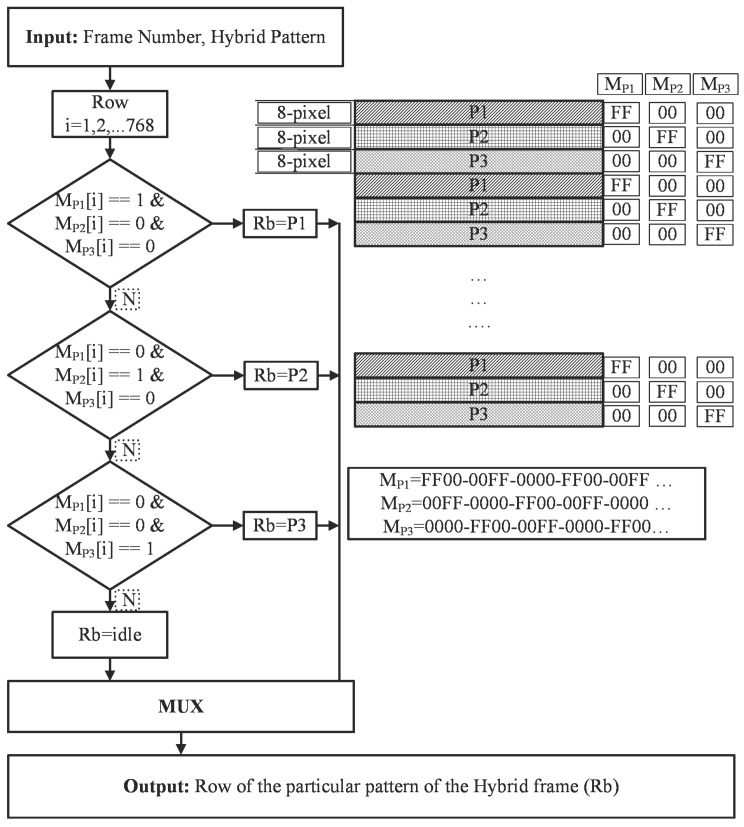
Spatial pattern generation module.

The combination of temporal and spatial patterns is known as hybrid patterns. Temporal patterns fail to capture the moving objects as the stripe cannot be decoded correctly due to the large scanning time of the scene. Hybrid patterns are required for moving objects where a high scan speed is required [38,64]. In this framework, we also implemented hybrid patterns to project on the scene to capture the high scan speed applications. For hybrid patterns, the more completed part is the spatial patterns and the temporal patterns are projected by same as GCI and HOC pattern generation module. The spatial pattern has a particular characteristic which consists of an 8 × 8 pixel block, which means that the eight rows have the same values and is repeated after 24 pixels, so there are actually three different rows which are assigned to the buffer and are represented as P1, P2, and P3. The mechanism by which those are projected vertically is presented by M_P1_, M_P2_, and M_P3_, as shown in Figure 12. The sequence of occurrence of each row is stored in the “MPi” buffer (i = 1, 2, 3) and the pattern of each row is stored in “Pj” (j = 1, 2, 3). To project the spatial patterns, we only need six rows of information to record, and the mechanism to generate pixels for each row is shown in the block diagram. This method is unique and state of the art by which any kind of pattern can be projected through this technique.

### 3.6. Video Graphics Array Controller and Camera Trigger Module

**Figure 13:**
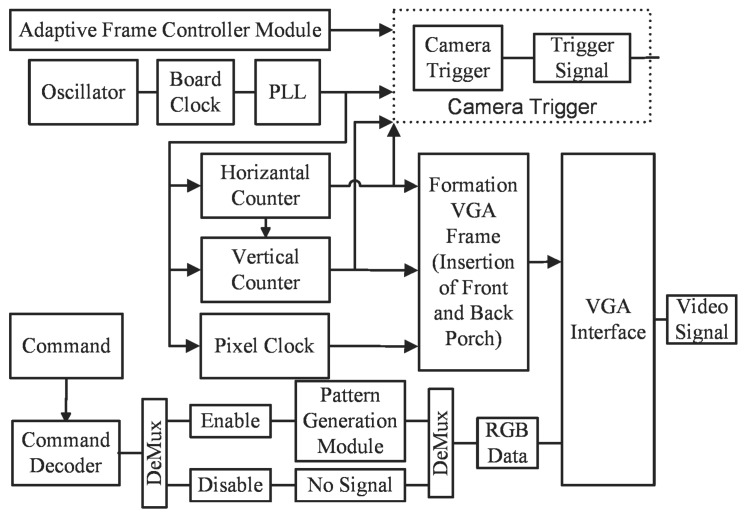
Block diagram of VGA controller and camera trigger module.

In FPGA implementation, four common modules for all kinds of pattern generation are the serial controller, adaptive frame rate mechanism, the VGA controller, and the trigger controller module. The trigger generation module accepts three inputs from two different modules, as shown in Figure 13. The pixel clock and vertical sync signal comes from the VGA controller module, and the frame number information comes from the adaptive frame controller module. The adaptive frame controller module decides the generation of trigger position based on the camera and projector exposure time, and the location is decided by the horizontal and vertical sync signal of the frame, which is controlled by the pixel clock of the VGA controller module. The VGA controller is the module that drives the projector and generates five signals, three colors and two synchronization signals: Red, Green, and Blue signals, and vertical and horizontal signals.

## 4. Experimental Results

Extensive experimentation was carried out to observe the general behavior of the implementation. We used two different cameras that were different in resolution and had different frame rates. The results are presented in terms of FPGA resources acquired by different techniques if implemented independently and in combination with other structured light pattern techniques. Performance of the hardware synchronization was better when compared to software synchronization [24,68]. Qualitative and quantitative analysis of the 3D scanning algorithm is also presented. The performance of two exposures is also presented, which shows that the capturing scene with different camera exposure time performed better than a single exposure time, and a dense and accurate point cloud was obtained by capturing the same pattern twice with different camera exposure settings.

### 4.1. Experimental Setup

**Figure 14:**
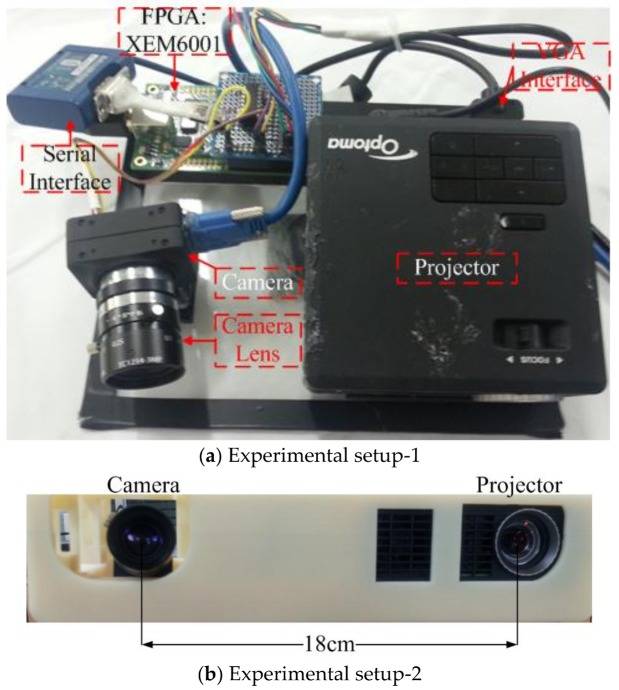
Experiments are performed on two different camera setups (**a**) Chameleon USB 3.0 camera with an Optoma ML 750 commercial projector; and (**b**) Flea3 FireWire with an Optoma ML 750 projector.

Two experimental setups are shown in Figure 14. Patterns were projected through the Optoma ML 750 [69], which is compact in size with an LED light source of 700 Lumen. Three different patterns were projected with pattern and projector resolutions of 1024 × 768. Two different configurations were made by using two cameras Chameleon3 [66] and Flea3 Firewire from Pointgrey, which are 1280 × 960@149 Hz and 640 × 480@120 Hz, respectively. An off the shelf XEM 6001 FPGA board from Opal Kelly was used to develop the controller module [70]. The Opal Kelly XEM6001 is an integration module based on a Xilinx Spartan-6 FPGA. XEM6001 features flexible clocking with a multi-output clock generator that can generate clock frequencies from 1 MHz to 150 MHz.

### 4.2. Field Programmable Gate Array Resources

FPGA resources are estimated by considering the individual implementation and combined implementation of three different structured light patterns. The results of the resources are shown in Table 1, which shows that implementing multiple patterns did not require a large number of different resources, which was evidence that a large framework can be implemented to project as many patterns by using the same hardware.

### 4.3. Qualitative Analysis for Codec Comparison

**Figure 15:**
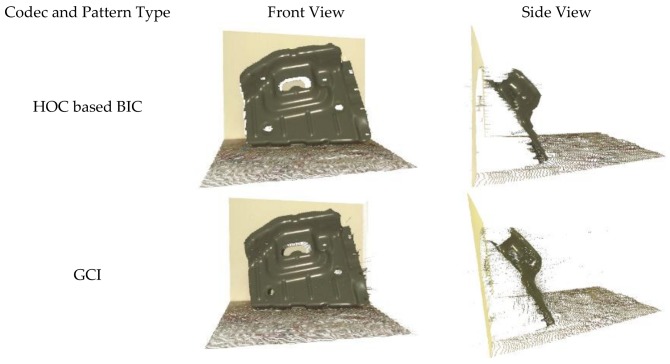

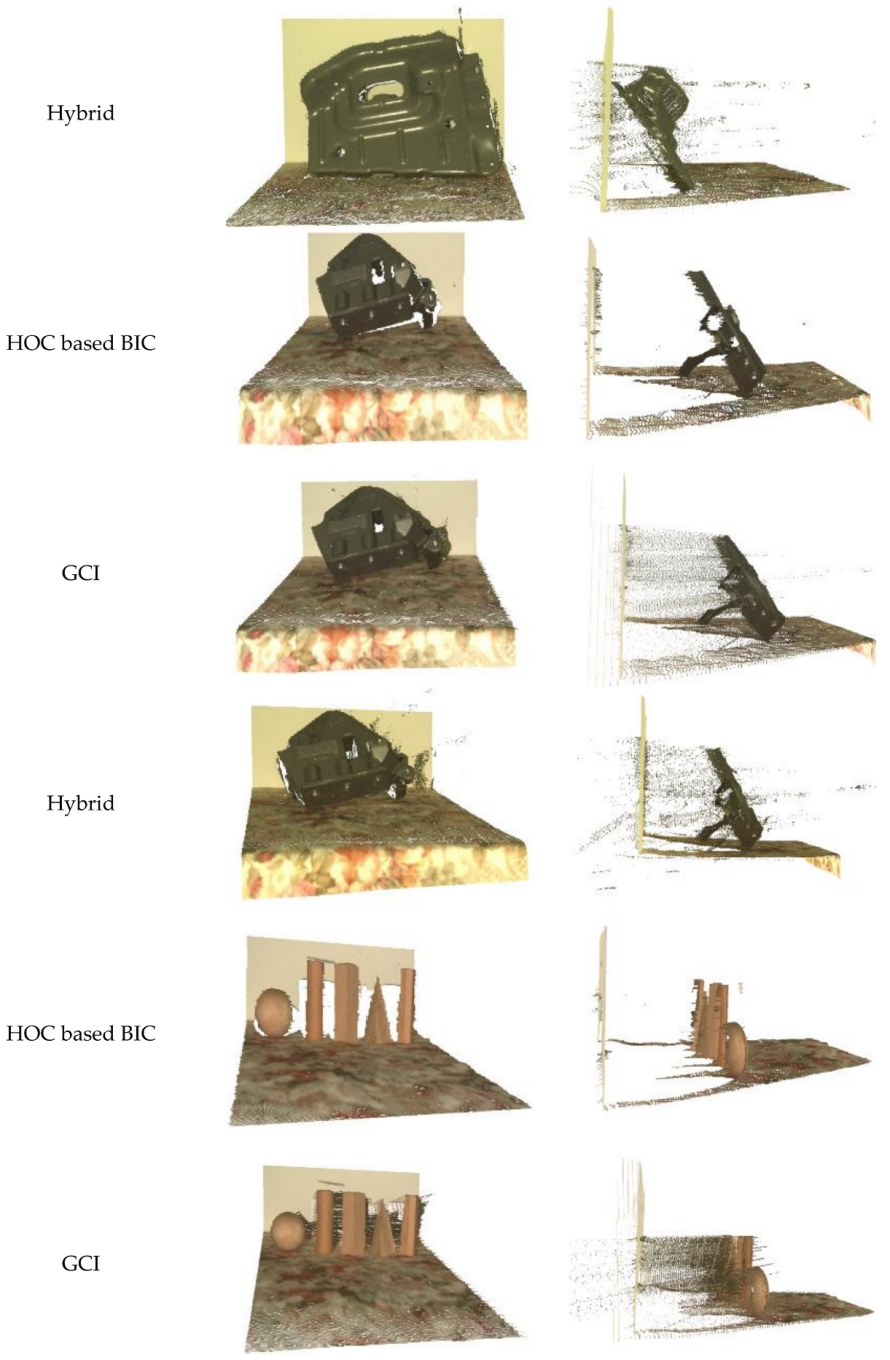

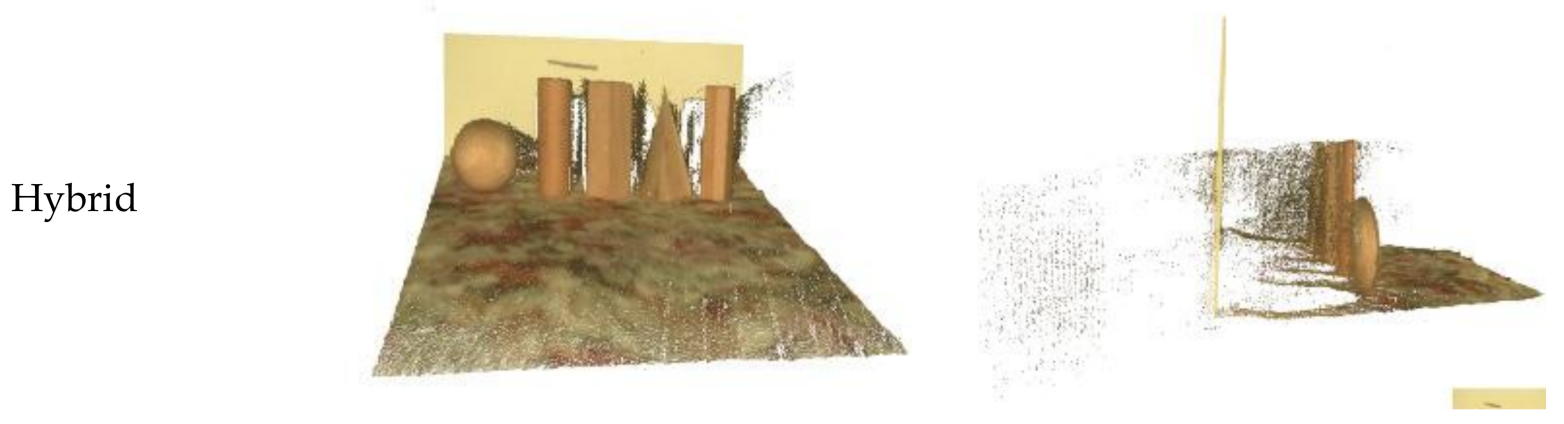
Qualitative evaluation for different structured light patterns projected through the FPGA.

Objects were captured by projecting the HOC, GCI, and Hybrid patterns by keeping all the parameters the same, such as exposure time and distance from the camera to the scene. The results were evidence that the point cloud obtained through the HOC patterns was clean and contained fewer outliers while the point cloud obtained through hybrid patterns was noisy and contained a lot of outliers. The side and top views of the 3D output are shown in Figure 15. Temporal codec such as HOC based BIC and GCI uses a larger number of projected patterns for a higher accuracy of 3D point cloud at the expense of scanning speed of hybrid codec. However, HOC based BIC provides even higher accuracy and fewer outliers than GCI because it adopts the so-called boundary inheritance in decoding for layer-wise boundary correspondence and correction [60].

### 4.4. Quantitative Analysis for Codec Comparison

**Figure 16:**
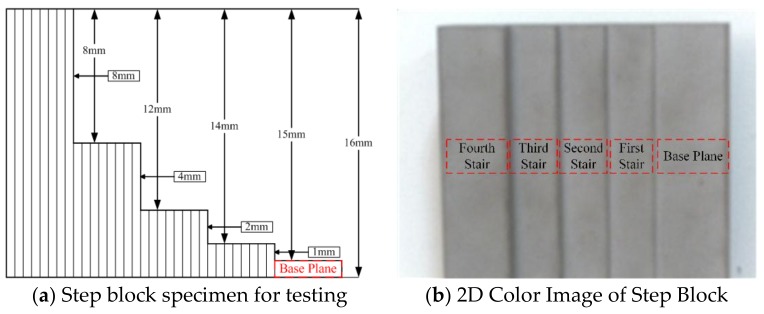
Precision measurement of the codec is performed through capturing the step block: (**a**) the specimen of the step block; and (**b**) the 2D color image of the captured step block.

A step block was captured at a distance of 100 cm from the scene to the camera. The precision for each step was calculated as discussed in [71]. The point cloud from each step surface is fitted onto a plane, the coefficients of which were estimated and the Euclidian distances between the fitted planes and those points were calculated. The results are presented in Table 2 and specimen of the step block is shown in Figure 16. A more detailed evaluation of the codec comparison has been previously discussed in [64].

These results show that the decoding of HOC patterns performed better than the other patterns.

### 4.5. Multi-Camera Exposure with Single Projector Exposure

**Figure 17:**
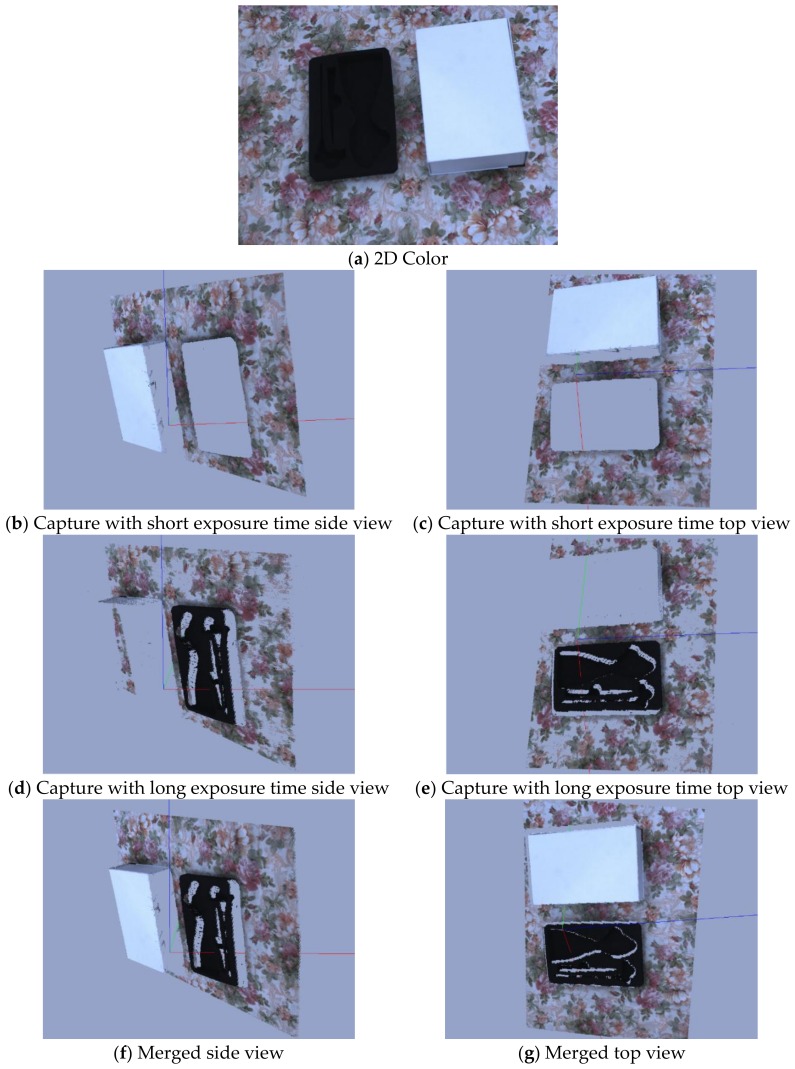
(**a**) 2D color image of the scene; (**b**,**c**) side and top view of 3D output captured with short exposure time, respectively; (**d**,**e**) side and top view of 3D output captured with long exposure time, respectively; and (**f**,**g**) side and top view of point cloud obtained after merging the point cloud obtained through short and long exposure times, respectively.

This is an example of a particular case where the scene is captured with two exposure settings: one with a short exposure and the other with a long exposure. As discussed earlier, the projector exposure time was sufficient to capture the scene with multiple camera exposure settings, but this can only be possible if the camera frame rate is higher than the projector screen refresh rate. The scene was captured with two exposure settings of the camera: 1 ms and 7 ms. The 3D output was obtained through two different exposure settings, as shown in Figure 17. These results clearly showed that the point cloud obtained through the single exposure time reduced the number of 3D points, but, if it was captured with two different exposure settings, the point cloud was denser and more accurate. With a short exposure time, highly reflective surfaces can be captured and with a longer exposure time, surfaces with low reflectivity can be captured. These results clearly indicate that objects with mixed reflective characteristics cannot be captured with a single exposure. 

#### 4.5.1. Qualitative Analysis

**Figure 18:**
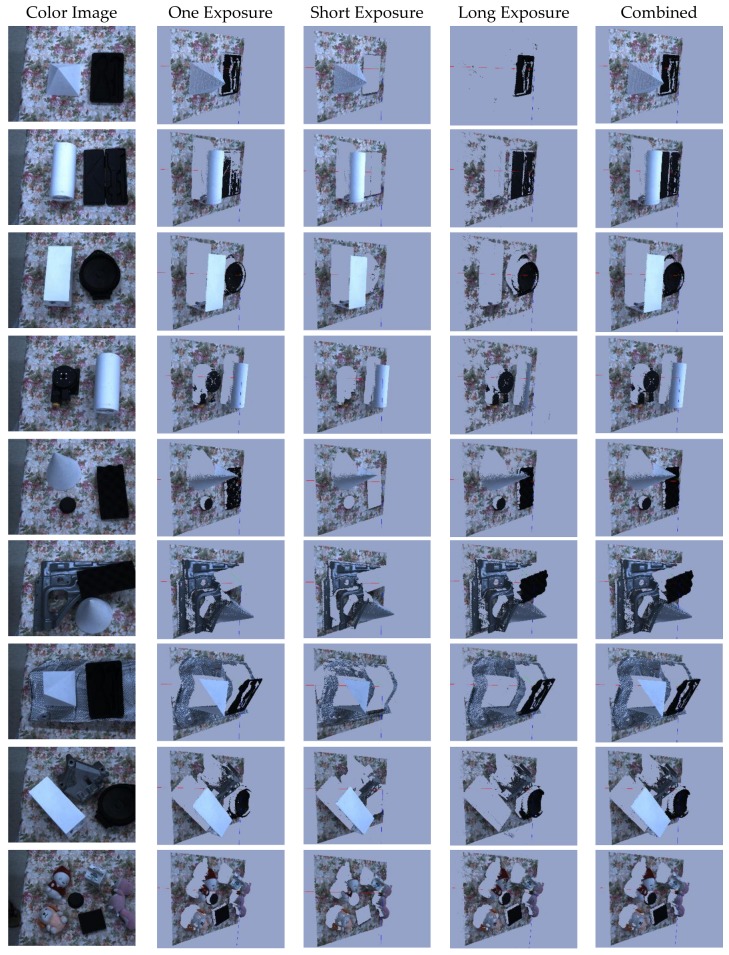

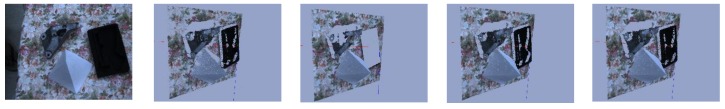
2D color image and side of 3D point cloud obtained by using one optimal exposure and multi-exposure capture within the single projection.

Objects with distinct reflectance characteristics are captured with single and multi-camera exposure are captured and presented in Figure 18. Fewer 3D points are generated from the single exposure which is evidence that one camera exposure is not enough to obtain a good quality point cloud. Point cloud obtained through the multi-exposure captured with short and long camera exposure settings are also presented. These results clearly show that single camera exposure settings are not enough to obtain good quality point clouds in the presence of surfaces with large reflectance variations. In this experiment, we obtained the number of 3D points by capturing the frame twice by triggering the camera twice within the single projector exposure.

#### 4.5.2. Quantitative Analysis

Several 3D points obtained through the single and multi-exposure capture are presented in Table 3. Increase in the number of 3D points is up to 31% by capturing the same frame twice, which shows that the proposed framework can be used to capture a scene which contains surfaces with large reflectance variations.

## 5. Discussion

A framework is proposed in this paper for an adaptive rate multiple pattern projection to capture objects with large reflectance variation for different scanning applications. The results show that the framework can synchronize the camera and projector system for mismatched frame rate and screen refresh rate, respectively. The whole platform can be used to project different kinds of patterns to obtain the point cloud for different scanning applications, e.g., hybrid patterns can be used to capture moving objects and HOC or GCI can be used to obtain dense and accurate point clouds of stationary objects. Available FPGA resources are sufficient to implement other patterns for different applications, which is evidence that a single platform could be used for different scanning applications without changing the architecture of the structured light 3D camera system. The adaptive frame rate pattern projection mechanism is available for all kinds of patterns and is flexible enough for use with any machine vision camera and commercial projector. High-resolution cameras have a low frame rate that could be synchronized with a commercial video projector by projecting the patterns multiple times to obtain a good quality point cloud at maximum speed. Scan time is reduced to half by capturing the same scene with different camera exposure settings, which improves the quality of the point cloud by capturing the multi-frame during the same projection time without additional projection. No external memory is required, which minimizes the cost of the ASIC design and reduces the circuit size. Logic blocks for the implementation of the system are simple and size effective and do not require additional FPGA resources. By working in this way, more patterns can be incorporated to project under the same platform and the HDR technique can be used to reconstruct the 3D information for different exposure settings. The HDR technique can be implemented to capture 3D information by using adaptive frame rate pattern projection where an N number of exposure steps are required for an HDR image, where the exposure step size and the value of N depend upon the reflectance properties of the objects.

## Figures and Tables

**Table 1:** FPGA resources required for developing a framework for the individual and multiple types of patterns.

Resources	Pattern Type	Available	Used	% Age
Number of Slice Registers	HOC (V/H)	18,224	1290	7
	GCI (V/H)		445	2
	Hybrid		184	1
	Combined		1321	7
Number of Slice Look-up Table (LUT)	HOC (V/H)	9112	2005	22
	GCI (V/H)		682	7
	Hybrid		302	3
	Combined		2789	30
Fully used Look-up Table-Flip Flop pairs	HOC (V/H)	2051	1244 (2051)	60
	GCI (V/H)		398 (729)	54
	Hybrid		142 (344)	41
	Combined		1250 (2860)	43

**Table 2:** Accuracy measurement for different structured light patterns at a 100 cm distance from the camera to the object.

Standard Deviation of Error in mm				
Pattern	1st Stair	2nd Stair	3rd Stair	4th Stair
HOC based BIC	0.08	0.11	0.12	0.19
GCI	0.12	0.14	0.16	0.20
Hybrid	0.20	0.26	0.29	0.38

**Table 3:** Several 3D points for multi-capture with a single exposure.

One Exposure	Low Exposure	High Exposure	Combined	% Age
775,541	839,326	146,400	916,002	16.6
644,903	714,890	414,006	882,296	31.08
751,669	784,836	383,371	886,567	16.46
826,828	802,321	439,616	896,735	8.11
757,063	794,097	675,912	927,845	20.27
719,585	678,178	744,814	900,874	22.37
661,942	810,599	616,170	816,391	20.89
698,963	766,365	607,945	863,725	21.08
802,547	816,928	720,475	890,730	10.41
740,800	822,365	649,831	908,309	20.31

## References

[1] Geng J. (2011). Structured-light 3D surface imaging: A tutorial. Adv. Opt. Photonics.

[2] Dhond U.R., Aggarwal J.K. (1989). Structure from stereo—A review. IEEE Trans. Syst. Man. Cybern..

[3] Cui Y., Schuon S., Chan D., Thrun S., Theobalt C. 3D shape scanning with a time-of-flight camera. Proceedings of the 2010 IEEE Computer Society Conference on Computer Vision and Pattern Recognition.

[4] Gong Y., Zhang S. (2010). Ultrafast 3-D shape measurement with an off-the-shelf DLP projector. Opt. Express.

[5] Yau H.T., Yang T.J., Lin Y.K. (2016). Comparison of 3-D Printing and 5-axis Milling for the Production of Dental e-models from Intra-oral Scanning. Comput. Aided Des. Appl..

[6] Wissmann P., Schmitt R., Forster F. Fast and Accurate 3D Scanning Using Coded Phase Shifting and High Speed Pattern Projection. Proceedings of the 2011 International Conference on 3D Imaging, Modeling, Processing, Visualization and Transmission.

[7] Gupta M., Yin Q., Nayar S.K. Structured Light in Sunlight. Proceedings of the 2013 IEEE International Conference on Computer Vision.

[8] Ishii I., Koike T., Hao G., Takaki T. Fast 3D shape measurement using structured light projection for a one-directionally moving object. Proceedings of the 37th Annual Conference of the IEEE Industrial Electronics Society, IECON 2011.

[9] Lanman D., Taubin G. (2009). Build your own 3D scanner. ACM SIGGRAPH 2009 Courses on–SIGGRAPH ’09.

[10] Yan K., Sukthankar R. PCA-SIFT: A more distinctive representation for local image descriptors. Proceedings of the 2004 IEEE Computer Society Conference on Computer Vision and Pattern Recognition.

[11] Rublee E., Rabaud V., Konolige K., Bradski G. ORB: An efficient alternative to SIFT or SURF. Proceedings of the 2011 International Conference on Computer Vision.

[12] Zhang S., Hyun J.-S., Li B., Douglass M.R., Lee B.L. (2017). High-speed 3D imaging using digital binary defocusing method vs sinusoidal method. Emerging Digital Micromirror Device Based Systems and Applications IX.

[13] Microsoft 3D Scan with Kinect—Windows Hardware Dev Center. https://developer.microsoft.com/en-us/windows/hardware/3d-print/scanning-with-kinect.

[14] Kim M., Kim S., Park S., Choi M.-T., Kim M., Gomaa H. (2009). Service robot for the elderly. IEEE Robot. Autom. Mag..

[15] Kim J.-J., Hong S., Lee W., Kang S., Lee S., Atif M., Do H.M., Choi T.Y., Park D.I.L., Son Y. (2016). Modman: self-reconfigurable modular manipulation system for expansion of robot applicability. Advances in Cooperative Robotics.

[16] AT (Automation Technology Vision Sensors and Systems) 3D Inspection for Completeness or Coplanarity Checks of BGAs. http://www.automationtechnology.de/cms/en/completeness-check-and-und-koplanaritaetspruefung-von-bgas/.

[17] Hansen K., Pedersen J., Solund T., Aanaes H., Kraft D. A Structured Light Scanner for Hyper Flexible Industrial Automation. Proceedings of the 2014 2nd International Conference on 3D Vision.

[18] Zanuttigh P., Marin G., Dal Mutto C., Dominio F., Minto L., Cortelazzo G.M. (2016). Operating Principles of Structured Light Depth Cameras. Time-of-Flight and Structured Light Depth Cameras.

[19] Buchón-Moragues F., Bravo J., Ferri M., Redondo J., Sánchez-Pérez J. (2016). Application of Structured Light System Technique for Authentication of Wooden Panel Paintings. Sensors.

[20] Wang Z. (2015). An Imaging and Measurement System for Robust Reconstruction of Weld Pool During Arc Welding. IEEE Trans. Ind. Electron..

[21] Lee S., Wei L., Naguib A.M. Adaptive Bayesian recognition and pose estimation of 3D industrial objects with optimal feature selection. Proceedings of the 2016 IEEE International Symposium on Assembly and Manufacturing (ISAM).

[22] Sukhan L., Jongmoo C., Seungsub O., Jaehyuk R., Jungrae P. A real-time 3D IR camera based on hierarchical orthogonal coding. Proceedings of the 2006 IEEE International Conference on Robotics and Automation.

[23] Wang Y., Liu K., Hao Q., Lau D.L., Hassebrook L.G. (2011). Period Coded Phase Shifting Strategy for Real–time 3-D Structured Light Illumination. IEEE Trans. Image Process..

[24] Atif M., Lee S. (2017). FPGA Based Pattern Generation and Synchonization for High Speed Structured Light 3D Camera. TELKOMNIKA Telecommun. Comput. Electron. Control..

[25] Photonics K. LC3000 Pro Projector. http://www.keynotephotonics.com/featured-products/lc3000/.

[26] Zhang S., Yau S.-T. (2006). High-resolution, real-time 3D absolute coordinate measurement based on a phase-shifting method. Opt. Express.

[27] Ryu M., Kim D., Lee S., Lee J. Optimal Exposure Estimation in the Image for Structured Light System. Proceedings of the ICMIT 2007: Mechatronics, MEMS, and Smart Materials.

[28] Ekstrand L., Zhang S. (2011). Autoexposure for three-dimensional shape measurement using a digital-light-processing projector. Opt. Eng..

[29] Yau S.-T., Yau S.-T. (2009). High dynamic range scanning technique. Opt. Eng..

[30] Weinmann M., Schwartz C., Ruiters R., Klein R. A Multi-camera, Multi-projector Super-Resolution Framework for Structured Light. Proceedings of the 2011 International Conference on 3D Imaging, Modeling, Processing, Visualization and Transmission.

[31] Petković T., Pribanić T., Djonlić M., D’apuzzo N. Software Synchronization of Projector and Camera for Structured Light 3D Body Scanning. Proceedings of the 7th International Conference on 3D Body Scanning Technologies.

[32] Bellis S.J., Marnane W.P. (2000). A CORDIC Arctangent FPGA Implementation for a High-Speed 3D-Camera System.

[33] Jongenelen A.P.P., Bailey D.G., Payne A.D., Carnegie D.A., Dorrington A.A. (2012). Efficient FPGA implementation of homodyne-based time-of-flight range imaging. J. Real-Time Image Process..

[34] Hong B.-J., Park C.-O., Seo N.-S., Cho J.-D. (2012). A Real-time Compact Structured-light based Range Sensing System. J. Semicond. Technol. Sci..

[35] Feng S., Chen Q., Zuo C., Tao T., Hu Y., Asundi A. (2017). Motion-oriented high speed 3-D measurements by binocular fringe projection using binary aperiodic patterns. Opt. Express.

[36] Sagawa R., Ota Y., Yagi Y., Furukawa R., Asada N., Kawasaki H. Dense 3D reconstruction method using a single pattern for fast moving object. Proceedings of the 2009 IEEE 12th International Conference on Computer Vision.

[37] Hall-Holt O., Rusinkiewicz S. Stripe boundary codes for real-time structured-light range scanning of moving objects. Proceedings of the 8th IEEE International Conference on Computer Vision, ICCV 2001.

[38] Zhang Y., Xiong Z., Yang Z., Wu F. (2014). Real-Time Scalable Depth Sensing With Hybrid Structured Light Illumination. IEEE Trans. Image Process..

[39] Sansoni G., Trebeschi M., Docchio F. (2009). State-of-The-Art and Applications of 3D Imaging Sensors in Industry, Cultural Heritage, Medicine, and Criminal Investigation. Sensors.

[40] Pagès J., Salvi J., Collewet C., Forest J. (2005). Optimised De Bruijn patterns for one-shot shape acquisition. Image Vis. Comput..

[41] Barone S., Paoli A., Razionale A. (2013). A Coded Structured Light System Based on Primary Color Stripe Projection and Monochrome Imaging. Sensors.

[42] Geng J., Douglass M.R., Oden P.I. (2011). DLP-Based Structured Light 3D Imaging Technologies and Applications.

[43] Konolige K. Projected texture stereo. Proceedings of the 2010 IEEE International Conference on Robotics and Automation.

[44] Lee S., Choi J., Kim D., Na J., Seungsub O. Signal Separation Coding for Robust Depth Imaging Based on Structured Light. Proceedings of the Proceedings of the 2005 IEEE International Conference on Robotics and Automation.

[45] Guehring J., El-Hakim S.F., Gruen A. (2000). Dense 3D Surface Acquisition by Structured Light Using Off-The-Shelf Components.

[46] Ishii I., Yamamoto K., Doi K., Tsuji T. High-speed 3D image acquisition using coded structured light projection. Proceedings of the 2007 IEEE/RSJ International Conference on Intelligent Robots and Systems.

[47] Nguyen T.T., Slaughter D.C., Max N., Maloof J.N., Sinha N. (2015). Structured light-based 3D reconstruction system for plants. Sensors.

[48] Wu D., Chen T., Li A. (2016). A High Precision Approach to Calibrate a Structured Light Vision Sensor in a Robot-Based Three-Dimensional Measurement System. Sensors.

[49] Zhan D., Yu L., Xiao J., Chen T. (2015). Multi-Camera and Structured-Light Vision System (MSVS) for Dynamic High-Accuracy 3D Measurements of Railway Tunnels. Sensors.

[50] Bräuer-Burchardt C., Brahm A., Heist S., Dietrich P., Kühmstedt P., Notni G. (2017). Accurate 3D Face and Body Scanning Using an Irritation-Free Pattern Projection System. Proceedings.

[51] Park J., Lee S., Lee I. (2009). Precise 3D Lug Pose Detection Sensor for Automatic Robot Welding Using a Structured-Light Vision System. Sensors.

[52] Ahn J., Park A., Kim J., Lee B., Eom J. (2017). Development of Three-Dimensional Dental Scanning Apparatus Using Structured Illumination. Sensors.

[53] Bräuer-Burchardt C., Heinze M., Schmidt I., Kühmstedt P., Notni G. (2016). Underwater 3D Surface Measurement Using Fringe Projection Based Scanning Devices. Sensors.

[54] Lee S., Atif M., Han K. Stand-Alone Hnad-Eye 3D Camera for Smart Modular Manipulator. Proceedings of the IEEE/RSJ IROS Workshop on Robot Modularity.

[55] Kang S., Kim J.-J., Hong S., Lee W., Lee S., Atif M., Do H.M., Choi T.Y., Park D.I., Son Y. MODMAN: Modular Manipulation System with Self-Reconfigurable Perception and Motion Engines for Easy Task Adaptation. Proceedings of the IEEE/RSJ IROS Workshop on Robot Modularity.

[56] Anwar I., Lee S. High performance stand-alone structured light 3D camera for smart manipulators. Proceedings of the 2017 14th International Conference on Ubiquitous Robots and Ambient Intelligence (URAI).

[57] Olaya E.J., Berry F., Mezouar Y. A robotic structured light camera. Proceedings of the 2014 IEEE/ASME International Conference on Advanced Intelligent Mechatronics.

[58] Nguyen H., Nguyen D., Wang Z., Kieu H., Le M. (2015). Real-time, high-accuracy 3D imaging and shape measurement. Appl. Opt..

[59] Salvi J., Fernandez S., Pribanic T., Llado X. (2010). A state of the art in structured light patterns for surface profilometry. Pattern Recognit..

[60] Bui L.Q., Lee S. (2013). Boundary Inheritance Codec for high-accuracy structured light three-dimensional reconstruction with comparative performance evaluation. Appl. Opt..

[61] Lee S., Bui L.Q. (2011). Accurate estimation of the boundaries of a structured light pattern. J. Opt. Soc. Am. A.

[62] Atif M., Lee S. Boundary based shade detection. Proceedings of the 2016 IEEE International Conference on Multisensor Fusion and Integration for Intelligent Systems (MFI).

[63] Dung H.T.N., Lee S. Outlier removal based on boundary order and shade information in structured light 3D camera. Proceedings of the 2015 IEEE 7th International Conference on Cybernetics and Intelligent Systems (CIS) and IEEE Conference on Robotics, Automation and Mechatronics (RAM).

[64] Bui Quang Lam A Boundary Inheritance Codec for Structured Light Based Depth Imaging System.. http://dcollection.skku.edu/jsp/common/DcLoOrgPer.jsp?sItemId=000000050032.

[65] Point Grey Research Grasshopper3 USB3 Vision Cameras for Industrial, Life Science, Traffic, and Security Applications. https://www.ptgrey.com/grasshopper3-usb3-vision-cameras.

[66] Point Grey Chameleon3 Board Level USB3 Vision Cameras for Industrial, Life Science, Traffic, and Security Applications. https://www.ptgrey.com/chameleon3-usb3-vision-cameras.

[67] Scharstein D., Szeliski R. High-accuracy stereo depth maps using structured light. Proceedings of the 2003 IEEE Computer Society Conference on Computer Vision and Pattern Recognition.

[68] Atif M., Lee S. Adaptive frame rate pattern projection for structured light 3D camera system. Proceedings of the 2017 IEEE International Conference on Multisensor Fusion and Integration for Intelligent Systems (MFI).

[69] Optoma ML750 DLP WXGA Business Projector: Optoma. https://www.optomausa.com/projectorproduct/ml750.

[70] XEM6001—Opal Kelly. https://www.opalkelly.com/products/xem6001/.

[71] Khoshelham K., Elberink S.O. (2012). Accuracy and Resolution of Kinect Depth Data for Indoor Mapping Applications. Sensors.

